# ESPO AND SOCIAL RESEARCH, POLICY, AND PRACTICE SECTION SYMPOSIUM: WHAT AROUND THE PERSON MATTERS: REIMAGINING ENVIRONMENTAL JUSTICE AND AGING

**DOI:** 10.1093/geroni/igac059.552

**Published:** 2022-12-20

**Authors:** Kexin Yu, Sarah Dys

## Abstract

From Lawton’s seminal Ecological Theory of Aging (ETA) to the recent development of Wahl’s COntext Dynamics of Aging (CODA), conceptual and empirical work has repeatedly shown that the living environments fundamentally influence health and wellbeing in later life. The CODA framework posits five correlated contexts that can predict developmental outcomes in aging: physical, social, service, socioeconomic, and technological contexts. The ongoing COVID-19 pandemic further manifested the lack of environmental justice for marginalized and minoritized older adults, calling for reflection on the paradigm for ecological aging research, practice, and actionable policymaking. This SRPP/ESPO symposium featured emerging and established scholars’ work that shed light on reimagining environmental justice for older adults with diverse abilities, backgrounds, and resources. Panelists will share stories of their professional development journeys, highlighting empirical evidence with under-researched, systematically excluded populations and examining new directions in aging and environment research. Topics include accessible and affordable housing, built environment, neighborhood contexts, conceptualizing the community, and the experiences of those most likely to bear the burden of precarious housing in later life. This symposium will hold the space for emerging scholars to learn and discuss short- and long-term practice and policy priorities for promoting environmental justice for older adults and provide tools to conceptualize their research informed through ecological and equity-centered perspectives.

